# The efficacy and toxicity of cabazitaxel for treatment of docetaxel-resistant prostate cancer correlating with the initial doses in Japanese patients

**DOI:** 10.1186/s12885-019-5342-9

**Published:** 2019-02-15

**Authors:** Naoki Terada, Toshiyuki Kamoto, Hiromasa Tsukino, Shoichiro Mukai, Shusuke Akamatsu, Takahiro Inoue, Osamu Ogawa, Shintaro Narita, Tomonori Habuchi, Shinichi Yamashita, Koji Mitsuzuka, Yoichi Arai, Shuya Kandori, Takahiro Kojima, Hiroyuki Nishiyama, Yoshiaki Kawamura, Yuki Shimizu, Toshiro Terachi, Motohiko Sugi, Hidefumi Kinoshita, Tadashi Matsuda, Yusuke Yamada, Shingo Yamamoto, Hiromi Hirama, Mikio Sugimoto, Yoshiyuki Kakehi, Toshihiko Sakurai, Norihiko Tsuchiya

**Affiliations:** 1 Department of Urology, Miyazaki University, Miyazaki, Japan; 2 Department of Urology, Kyoto University, Kyoto, Japan; 3 Department of Urology, Akita University, Akita, Japan; 4 Department of Urology, Tohoku University, Sendai, Japan; 5 Department of Urology, Tsukuba University, Tsukuba, Japan; 6 Department of Urology, Tokai University, Isehara, Japan; 7 Department of Urology and Andrology, Kansai Medical University, Osaka, Japan; 8 Department of Urology, Hyogo Medical University, Nishinomiya, Japan; 9 Department of Urology, Kagawa University, Takamatsu, Japan; 10 Department of Urology, Yamagata University, 2-2-2, Iida-nishi, Yamagata, 990-9585 Japan

**Keywords:** Prostate cancer, Cabazitaxel, Dosage, Efficacy, Toxicity, Predictive factors

## Abstract

**Background:**

We analyzed the efficacy and toxicity of cabazitaxel (CBZ) at high and low initial doses in Japanese patients with docetaxel-resistant castration-resistant prostate cancer (CRPC).

**Methods:**

We retrospectively evaluated 118 patients who received CBZ for docetaxel-resistant CRPC in 10 university hospitals in Japan between 2014 and 2016. The rate of decrease of prostate-specific antigen (PSA), adverse events, progression-free survival (PFS), and overall survival (OS) were compared between patients receiving initially high (≥22.5 mg/m^2^, *n* = 36) and low (≤20 mg/m^2^, *n* = 80) CBZ doses. Factors associated with survival and grade 4 neutropenia were evaluated.

**Results:**

PSA values decreased by > 50% in 22 patients (19%), with a higher frequency in the high-dose group than in the low-dose group (29 and 14%, *P* = 0.073). The median PFS time for the all-patient, high- and low-dose groups was 2.8 months (95% confidence interval [CI] 1.9–4.4), 2.1 months (1.2–5.5), and 3.0 months (2.0–4.4), respectively (*P* = 0.904). The median OS times were 16.3 months (95% CI 9.7–30.9), 30.9 months (11.8–47.4), and 10.2 months (8.6–20), respectively (*P* = 0.020). In multivariate analyses, PFS was significantly associated with existing bone metastasis at diagnosis (*P* = 0.005) and OS with PSA > 100 ng/ml (*P* = 0.007), hemoglobin < 12 g/dl (*P* = 0.030), and low initial CBZ dose (*P* = 0.030). Grade 4 neutropenia occurred in 53 patients (45%) and was associated with a low CBZ dose (hazard ratio 0.21, 95% CI 0.08–0.59, *P* = 0.002).

**Conclusions:**

CBZ at a higher initial dose may have similar response rate and response duration, but longer survival duration after treatment with higher toxicity than a lower initial dose for docetaxel-resistant CRPC in Japanese patients.

## Background

Prostate cancer is the second leading cause of cancer-related death in men in the USA [1] and its incidence in Japan is increasing rapidly [2]. Between 10 and 15% of patients present with advanced disease and receive hormone therapy as their initial treatment. However, most cases acquire therapy resistance within 2 years and then progress to castration-resistant prostate cancer (CRPC) [3]. In recent years, an increasing number of effective systemic therapies for metastatic CRPC have become available, including novel hormone treatments and taxane chemotherapies. Docetaxel was shown in 2004 to prolong the survival of patients with CRPC and was previously the only approved treatment [4]. Cabazitaxel (CBZ) is a taxane drug with activity against docetaxel-resistant cancers [5]. Overall survival (OS) of men with metastatic CRPC who had progressed after docetaxel-based chemotherapy was better after treatment with CBZ plus prednisone compared with mitoxantrone plus prednisone [6]. Based primarily on the results of that study [6], 25 mg/m^2^ CBZ in combination with prednisone was approved for the treatment of CRPC patients previously treated with docetaxel. Subsequent reports suggested that 20 mg/m^2^ CBZ might decrease myelotoxicity without altering efficacy [7]. However, the association between CBZ dose, efficacy, and toxicity has not been evaluated in Japanese patients with CRPC. The aim of this retrospective multi-institutional study was to evaluate the efficacy and toxicity of CBZ in correlation to the initial doses in Japanese patients with docetaxel-resistant CRPC.

## Methods

### Study population

This was a retrospective study of 118 patients who received CBZ for docetaxel-resistant CRPC at 10 university and satellite hospitals in Japan between 2014 and 2016. The study was approved by the institutional review board of each university. All patients had pathologically proven adenocarcinoma of the prostate that was progressing according to prostate-specific antigen (PSA) concentrations or radiographic criteria, despite androgen blockade therapy. The data were retrospectively obtained from medical records. CRPC was defined as either an increase in PSA level of > 25% relative to the nadir PSA value or radiological progression after initial hormonal therapy. All CRPC patients had received docetaxel before CBZ treatment. The criteria for decision making about CBZ doses was different between institutions and not determined. The rate of PSA decrease, incidence of adverse events, and duration of progression-free survival (PFS) and OS were compared between patients who received a first course of CBZ treatment of 25 or 22.5 mg/m^2^ (the high-dose group, *n* = 37) or 20 or 15 mg/m^2^ (the low-dose group, *n* = 81). The relative dose intensity (RDI) of CBZ was calculated as the ratio of delivered dose intensity (mg/m^2^/week) to the planned dose intensity (25 mg/m^2^, every 3 weeks) in the first three cycles of CBZ treatment. PFS was defined as the time from initiation of CBZ to an increase in PSA value > 25% relative to the nadir PSA value, radiological progression, or death after CBZ treatment. OS was defined as the time from initiation of CBZ treatment to death from any cause. The type and incidence of adverse events were also evaluated based on the laboratory data and medical records.

### Statistical analysis

The data are presented as medians and ranges. PFS and OS times were estimated using the Kaplan–Meier method and compared using the log-rank test. Associations between clinicopathological variables and prognosis were assessed using univariate and multivariate Cox’s proportional hazards regression models. The median values were used as cut-off values of the subgroups. Associations with adverse events were examined using multivariate logistic regression analysis. All statistical analyses were performed with EZR (Saitama Medical Center, Jichi Medical University, Saitama Japan), which is a graphical user interface for R (R Foundation for Statistical Computing, Vienna, Austria). *P* values of < 0.05 were considered statistically significant.

## Results

In total, 118 patients were treated with CBZ. Of these, 37 (31%) were in the high-dose group (30 received 25 mg/m^2^, 7 received 22.5 mg/m^2^ initially) and 81 (69%) were in the low-dose group (75 received 20 mg/m^2^, 6 received 15 mg/m^2^ initially). The clinicopathological characteristics for the patients in the high- and low-dose groups are shown in Table 1. Significantly more patients in the low-dose group than in the high-dose group had a performance status (PS) ≥1 (60 and 35%, respectively, *P* = 0.017), but there were no significant differences in other parameters. Patients were administered one dose of CBZ every 3 to 4 weeks (28 in 3 weeks and 90 in 4 weeks) and the median number of treatment cycles was 4 (range 1–33). During treatment, the CBZ dosage was decreased due to adverse events in 13 patients (35%) in the high-dose group and 6 patients (7%) in the low-dose group. Dosage was increased in 4 patients (5%) in the low-dose group and none in the high-dose group. RDI was calculated in 29 patients (78%) in the high-dose group and 56 patients (69%) in the low-dose group who received three or more cycles of CBZ treatment. The median RDI was 75% (60–100%) in the high-dose group and it was 60% (30–80%) in the low-dose group (*P* < 0.001) (Table 1). Prior treatments were surgery or radiation as local treatments (38/118, 32%), and abiraterone or enzalutamide (93/118, 79%). All patients had previously been treated with docetaxel (median 7 treatment cycles, range 1–61). In 97 patients (82%), pegfilgrastim was administered after the CBZ treatment.

**Table 1:** Patients background in high and low initial CBZ dose groups

Factors	Total (*n* = 118)	High (*n* = 37)	Low (*n* = 81)	*P (High* vs *Low)*
Median age at diagnosis (y)	65 (47–81)	65 (47–76)	66 (49–81)	0.087
Median PSA at diagnosis (ng/mL)	61.0 (5.0–21,114)	61.2 (3.2–21,114)	61.0 (5.0–9430)	0.840
GS at diagnosis≥9	70 (61%)	25 (68%)	45 (55%)	0.290
Metastatic status at diagnosis	No: 34 (29%)	No: 11 (30%)	No: 23 (28%)	0.528
	LN:15 (13%)	LN:5 (14%)	LN:10 (12%)	
	Bone< 6:37 (31%)	Bone< 6:8 (22%)	Bone< 6:29 (36%)	
	Bone> 5:24 (20%)	Bone> 5:10 (27%)	Bone> 5:14 (17%)	
	Visceral:8 (7%)	Visceral:3 (8%)	Visceral:5 (6%)	
Median age at CBZ start (y)	70 (48–88)	70 (48–79)	72 (50–88)	0.182
Median PSA at CBZ start (ng/ml)	75.2 (0.07–9586)	52.3 (3.44–1610)	78.0 (0.07–9586)	0.550
PS at CBZ start≥1	62 (53%)	13 (35%)	49 (60%)	0.017*
Median RDI of CBZ	60% (30–100%)	75% (60–100%)	60% (30–80%)	< 0.001*

*PS* performance status, *RDI* relative dose intensity *P < 0.05

**Fig. 1:**
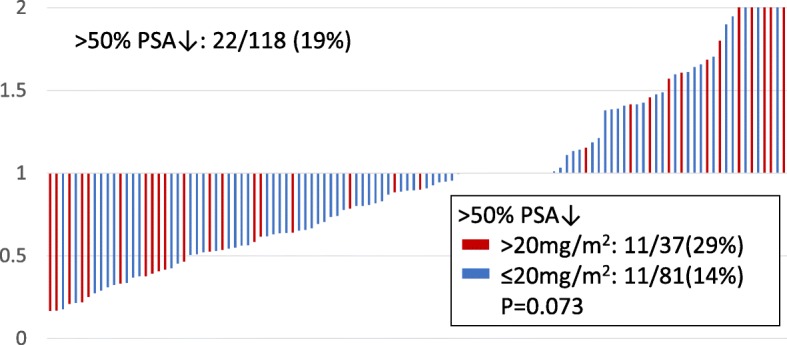
Waterfall plot of the PSA changes treated by cabazitaxel. The > 50% PSA decrease rate was 19% (22/118) in total. The PSA decrease rates in > 20 mg/m^2^ (red) and ≤ 20 mg/m^2^ (blue) were 29% (11/37) and 14% (11/81), respectively (*P* = 0.073)

**Fig. 2:**
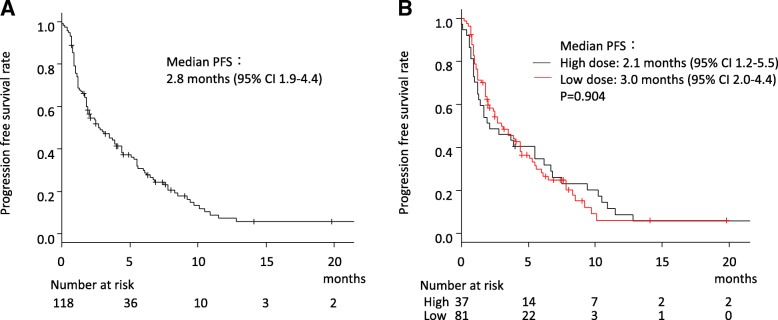
Progression-free survival (PFS) after cabazitaxel treatment. **a** PFS in the all-patient cohort (median survival 2.8 months, 95% CI 1.9–4.4). **b** PFS in the patients with the high initial CBZ dose (high-dose) group (median survival 2.1 months, 95% CI 1.2–5.5) and with the low initial CBZ dose (low-dose) group (median survival 3.0 months, 95% CI 2.0–4.4). *P* = 0.904 by log-rank test

The median follow-up duration was 8.2 months (range 1–50). PSA values decreased by > 50% in 22 patients (19%), with a higher frequency in the high-dose group (11/37, 29%) than in the low-dose group (11/81, 14%). However, this difference was not statistically significant (*P* = 0.073; Fig. 1). The median PFS time after CBZ treatment for all patients was 2.8 months (95% confidence interval [CI] 1.9–4.4; Fig. 2a) and there was no significant difference in PFS between the high- (median 2.1 months, 95% CI 1.2–5.5) and low-dose (median 3.0 months, 95% CI 2.0–4.4) groups (*P* = 0.904 by log-rank test; Fig. 2b). Univariate analysis of the association between PFS and age > 70 years, PS ≥1, total Gleason score ≥ 9, PSA > 100 ng/ml, hemoglobin < 12 g/dl, lactate dehydrogenase > 300 U/L, alkaline phosphatase > 350 U/L, presence of bone metastasis at diagnosis, time to CRPC < 1 year, previous docetaxel cycle < 10, previous abiraterone or enzalutamide treatment, initial low CBZ dose and RDI 60% or lower revealed that only the presence of bone metastasis at diagnosis was significantly associated with PFS (hazard ratio [HR] 1.55, 95% CI 1.03–2.34, *P* = 0.037), and this remained the only significantly associated factor after multivariate analysis including low initial CBZ dose (HR 1.03, 95% CI 1.03–2.37, *P* = 0.037; Table 2).

**Table 2:** Cox proportional hazard analyzes for PFS after CBZ treatment

	Univariate		Multivariate	
	Odds ratio (95% CI)	*P*	Odds ratio (95% CI)	*P*
Age (y) > 70	0.90 (0.60–1.35)	0.617		
PS ≥ 1	1.04 (0.69–1.56)	0.864		
GS ≥ 9	1.00 (0.65–1.53)	0.982		
PSA (ng/ml) > 100	0.93 (0.62–1.41)	0.743		
Hb (g/dl) < 12	1.245 (0.828–1.871)	0.292		
LDH (U/L) > 300	1.28 (0.81–2.02)	0.298		
ALP (U/L) > 350	0.93 (0.62–1.40)	0.739		
Bone meta at diagnosis	1.55 (1.03–2.34)	0.037*	1.03 (1.03–2.37)	0.037*
Time to CRPC (y) < 1	1.19 (0.79–1.79)	0.4		
DTX cycle< 10	1.40 (0.92–2.13)	0.121		
ABI or ENZA	0.73 (0.45–1.17)	0.184		
Low initial CBZ dose	1.03 (0.67–1.58)	0.905	0.95 (0.62–1.47)	0.832
RDI 60% or lower	0.86 (0.54–1.39)	0.545		

*PS* performance status, *DTX* docetaxel, *ABI* abiraterone, *ENZ* enzalutamide

*RDI* relative dose intensity, *P < 0.05

**Fig. 3:**
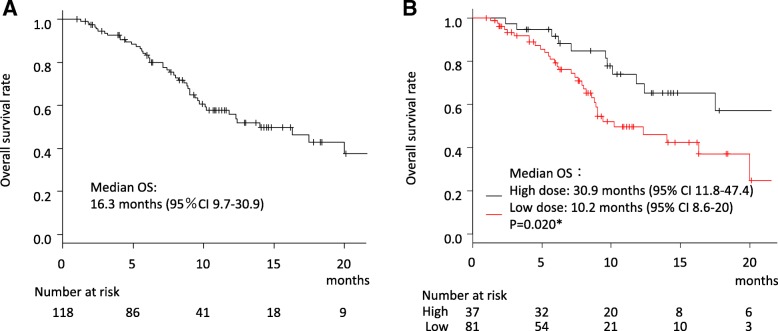
Overall survival (OS) after cabazitaxel treatment. **a** OS in the all-patient cohort (median survival 16.3 months, 95% CI 19.7–30.9). **b** OS in the patients with the high initial CBZ dose (high-dose) group (median survival 30.9 months, 95% CI 11.8–47.4) and with the low initial CBZ dose (low-dose) group (median survival 10.2 months, 95% CI 8.6–20). *P* = 0.020 by log-rank test

The median OS time after CBZ treatment was 16.3 months (95% CI 9.7–30.9; Fig. 3a). OS was significant longer in the high-dose group than the low-dose group (median 30.9 months, 95% CI 11.8–47.4 vs 10.2 months, 95% CI 8.6–20; *P* = 0.020 by log-rank test; Fig. 3b). Univariate Cox’s proportional hazard analysis identified significant association between OS and PS ≥1 (HR 3.14, 95% CI 1.63–6.04, *P* = 0.001), PSA > 100 ng/ml (HR 2.55, 95% CI 1.41–4.58, *P* = 0.002), hemoglobin < 12 g/dl (HR 1.95, 95% CI 1.03–3.72, *P* = 0.042), lactate dehydrogenase > 300 U/L (HR 2.03, 95% CI 1.10–3.74, *P* = 0.023), alkaline phosphatase > 350 U/L (HR 1.98, 95% CI 1.07–3.67, *P* = 0.03), initial low CBZ dose (HR 2.22, 95% CI 1.11–4.43, *P* = 0.023), and RDI 60% or lower (HR 2.76, 95% CI 1.24–6.17, *P* = 0.013). In multivariate analyses including these parameters, only the association with PSA > 100 ng/ml (HR 2.35, 95% CI 1.07–5.18, *P* = 0.034) remained significant. Then, using stepwise regression multivariate analyses, PSA > 100 ng/ml (HR 2.84, 95% CI 1.33–6.03, *P* = 0.007), hemoglobin < 12 g/dl (HR 2.50, 95% CI 1.09–5.73, *P* = 0.030) and low initial CBZ dose (HR 2.81, 95% CI 1.11–7.13, *P* = 0.030) were significantly correlated with OS (Table 3).

**Table 3:** Cox proportional hazard analyzes for OS after CBZ treatment

	Univariate		Multivariate		Multivariate (Stepwise)	
	Odds ratio (95% CI)	*P*	Odds ratio (95% CI)	*P*	Odds ratio (95% CI)	*P*
Age (y) > 70	1.14 (0.64–2.04)	0.654				
PS ≥ 1	3.14 (1.63–6.04)	0.001*	1.43 (0.55–3.73)	0.461		
GS ≥ 9	1.07 (0.58–2.00)	0.827				
PSA (ng/ml) > 100	2.55 (1.41–4.58)	0.002*	2.35 (1.07–5.18)	0.034*	2.84 (1.33–6.03)	0.007*
Hb (g/dl) < 12	1.95 (1.03–3.72)	0.042*	2.03 (0.80–5.15)	0.136	2.50 (1.09–5.73)	0.030*
LDH (U/L) > 300	2.03 (1.10–3.74)	0.023*	1.08 (0.45–2.60)	0.864		
ALP (U/L) > 350	1.98 (1.07–3.67)	0.030*	1.98 (0.83–4.69)	0.122		
Bone meta at diagnosis	1.00 (0.55–1.81)	0.998				
Time to CRPC (y) < 1	1.42 (0.79–2.54)	0.238				
DTX cycle< 10	1.81 (0.95–3.43)	0.071				
ABI or ENZA	1.66 (0.76–3.60)	0.201				
Low Initial CBZ dose	2.22 (1.11–4.43)	0.023*	2.15 (0.48–9.76)	0.32	2.81 (1.11–7.13)	0.030*
RDI 60% or lower	2.76 (1.24–6.17)	0.013*	1.26 (0.36–4.34)	0.717		

*PS* performance status, *DTX* docetaxel, *ABI* abiraterone, *ENZ* enzalutamide

RDI:relative dose intensity, *P < 0.05

The number of patients with CBZ-related adverse events is shown in Table 4. Thirteen patients (11%) terminated treatment due to adverse events. There were significant differences between the high and low CBZ dose groups in the incidence of grade 4 neutropenia (65 and 36%, respectively, *P* < 0.001) and grade 3 thrombocytopenia (16 and 4%, respectively, *P* = 0.026; Table 4). Univariate and multivariate logistic regression analyses of the association between grade 4 neutropenia and age, PS, docetaxel cycle, pegfilgrastim use, and low CBZ dose revealed that only low CBZ dose was significantly associated (univariate HR 0.21, 95% CI 0.08–0.52, *P* = < 0.001, multivariate HR 0.21, 95% CI 0.08–0.59, *P* = 0.002; Table 5).

**Table 4:** Number of adverse effects of CBZ treatment

	Total (*n* = 118)	High (*n* = 37)	Low (*n* = 81)	*P (High* vs *Low)*
Grade 4 neutropenia	53 (45%)	24 (65%)	29 (36%)	< 0.001*
Febrile neutropenia	30 (25%)	13 (35%)	17 (21%)	0.118
Grade 3 thrombocytopenia	9 (8%)	6 (16%)	3 (4%)	0.026*
Grade 3 nausea	17 (14%)	5 (14%)	12 (15%)	1
Grade 2 diarrhea	13 (11%)	4 (11%)	9 (11%)	1
Grade 2 malaise	19 (16%)	4 (11%)	16 (20%)	0.296
Pneumonia	5 (4%)	2 (5%)	3 (4%)	0.648

*P < 0.05

**Table 5:** Univariate and multivariate logistic regression analyses for the uccurence of grade 4 neutropenia

Factors	Number (%)	Univariate		Multivariate	
		Odds ratio (95% CI)	*P*	Odds ratio (95% CI)	*P*
Age (y) > 70	58 (49%)	1.45 (0.68–3.09)	0.340	1.99 (0.85–4.64)	0.113
PS ≥ 1	62 (53%)	0.55 (0.26–1.18)	0.126	0.71 (0.30–1.66)	0.425
DTX cycle< 10	72 (61%)	0.56 (0.26–1.12)	0.146	0.53 (0.22–1.25)	0.148
PEG GCSF use	97 (82%)	0.73 (0.27–2.02)	0.548	0.94 (0.28–3.18)	0.916
Low initial CBZ dose	81 (69%)	0.21 (0.08–0.52)	< 0.001*	0.21 (0.08–0.59)	0.002*

*PS* performance status, *DTX* docetaxel, PEG:pegfilgrastim, *P < 0.05

## Discussion

Based on the results of the phase III TROPIC trial, CBZ at 25 mg/m^2^ in combination with prednisone was approved in 2010 for the treatment of patients with CRPC previously treated with docetaxel. However, severe hematological toxicity was reported, and the possibility was raised that lower doses might be required to reduce myelotoxicity. This was tested in the phase III PROSELICA study, which evaluated the noninferiority and safety of 20 mg/m^2^ CBZ compared with 25 mg/m^2^. PSA response rates of > 50% were significantly higher in the 25 mg/m^2^ group (42.9%) than the 20 mg/m^2^ group (29.5%, *P* = 0.001). However, there was no significant difference in either the median PFS time (2.9 and 3.5 months in the 20 mg/m^2^ and 25 mg/m^2^ groups, HR 1.099, 95% CI 0.974–1.240) or median OS time (13.4 and 14.5 months in the 20 mg/m^2^ and 25 mg/m^2^ groups, HR 1.024, 95% CI 0.989–1.184) between the treatment groups. We found a similar PFS in the two treatment groups even though the PSA decrease rate tended to be lower in the low initial CBZ dose group. In contrast, the high-dose group had a significantly longer OS than did the low-dose group in univariate and multivariate Cox proportional hazard analyses. The RDI was significantly higher in the high-dose group. Based on these results, the initial high-dose CBZ has a potential to enhance the RDI, causing longer survival after treatment. However, our study was retrospective and the CBZ treatment protocol differed between institutes and physicians. The results might be influenced by some selection bias, which suggests that other patient characteristics were likely to explain the difference in OS. Moreover, the RDI of CBZ was smaller than 60% in many patients. In the real world, we had easily decreased the dose of CBZ because of high rate adverse events such as neutropenia. Based on the results of this study, we need to make efforts to increase the RDI so that CBZ can be used effectively. The appropriate dose of CBZ should be determined based on the disease status and characteristics in each patient.

In the PROSELICA study, grade 3 and 4 neutropenia was observed in 57.1 and 82.1% of patients treated with 20 mg/m^2^ and 25 mg/m^2^ CBZ, respectively, which supports the notion that a lower dose of CBZ may decrease the hematological toxicity rate [7]. In our study, grade 4 neutropenia was significantly less frequently observed in the low-dose group (36%) than in the high-dose group (65%; *P* < 0.001), which is similar to the results of the PROSELICA study. Moreover, grade 4 neutropenia was not significantly associated with age or PS in our study, which supports the possibility that low-dose CBZ could be safe even for older patients and/or those with low PS. Kosaka et al. reported that the presence of grade 3 or 4 neutropenia after CBZ treatment was significantly correlated with longer OS [8]. In our study, the presence of grade 4 neutropenia was correlated with longer OS after CBZ treatment (data not shown). These results indicated that the high-dose CBZ induced high-grade neutropenia associated with longer survival for docetaxel-resistant prostate cancer patients.

The PSA response rate in our study was 19%, which is significantly lower than those in the TROPIC and PROSELICA studies. The proportion of patients who received abiraterone or enzalutamide was also different between the studies; i.e., 0, 26, and 79% in TROPIC, PROSELICA, and our study, respectively. We previously reported that patients treated sequentially with abiraterone and enzalutamide showed a poorer response to the second than to the first treatment [9]. Therefore, one possible explanation for the lower PSA response rate in our study is that a much higher proportion of our patients than the TROPIC or PROSELICA patients had previously received abiraterone or enzalutamide.

Elucidating an appropriate treatment sequence using novel hormone treatments including abiraterone and enzalutamide and taxane chemotherapies including docetaxel and CBZ is important for maximizing clinical benefit in CRPC patients. We previously identified factors predicting efficacy in CRPC patients treated with enzalutamide [10]. We found that Gleason score, time to CRPC, PS, and previous steroid treatment were significantly associated with shorter PFS. In contrast, in the present study, only existing bone metastasis at diagnosis associated with PFS. By using these known baseline clinical parameters, we can predict the efficacy of novel treatments for prostate cancer. However, they are not perfect for selecting the best treatment sequence. Achieving precision medicine will require more precise tissue- or liquid-based biomarkers beyond these clinical parameters [11].

There are several limitations to our study. The number of patients is too small to allow precise statistical analyses. In addition, the study was retrospective and lacked a control group, and the treatment protocol differed between institutions. Moreover, we missed some prognostic parameters of the patients, such as metastatic status at the time of CBZ treatment, the efficacy of previous docetaxel, abiraterone, and enzalutamide, and the usage of bone-modifying agents, such as bisphosphonates, denosumab, and alpharadin. However, to our knowledge, this is the largest study (118 patients) of the treatment efficacy and toxicity of CBZ in Japanese patients to date. Further prospective studies are needed to determine the optimal dose of CBZ for Japanese subjects with CRPC.

## Conclusions

Existing bone metastasis and high PSA levels predicted shorter PFS and OS, respectively, after CBZ treatment. CBZ at higher initial doses may have similar response rate and response duration, but longer survival duration after treatment with higher toxicity than lower initial doses for docetaxel-resistant CRPC in Japanese patients.

## Abbreviations

CBZ: Cabazitaxel
CI: Confidence interval
CRPC: Castration-resistant prostate cancer
OS: Overall survival
PFS: Progression-free survival
PS: Performance status
PSA: Prostate-specific antigen
